# Identification of a Novel Signature Based on Ferritinophagy-Related Genes to Predict Prognosis in Lung Adenocarcinoma: Focus on AHNAK2

**DOI:** 10.3390/bioengineering11111070

**Published:** 2024-10-26

**Authors:** Liangjiang Xia, Haitao Ma

**Affiliations:** Department of Thoracic Surgery, The First Affiliated Hospital of Soochow University, 899 Pinghai Street, Suzhou 215006, China; 20214032023@stu.suda.edu.cn

**Keywords:** lung adenocarcinoma, ferritinophagy, prognostic signature, immune landscape, therapeutic response, AHNAK2

## Abstract

Background: Lung adenocarcinoma (LUAD) accounts for over 40% of all non-small cell lung cancer (NSCLC) cases and continues to be difficult to treat despite advancements in diagnostics and therapies. Ferritinophagy, a newly recognized autophagy process linked to ferroptosis, has been associated with LUAD development. Recent studies have shown a dysregulation of genes related to ferritinophagy in LUAD, indicating its potential as a therapeutic target. Methods: We constructed a predictive model using seven genes associated with ferritinophagy. The model’s accuracy was evaluated across three independent gene expression datasets. We analyzed the biological functions, immune environment, mutations, and drug sensitivities in groups with high and low risk. Utilizing a single-cell sequencing (scRNA-seq) dataset, we confirmed the expression of the model genes and identified a subtype of epithelial cells expressing AHNAK2. We further investigated the impact of the ferritinophagy-related gene AHNAK2 on LUAD cell proliferation, invasion, migration, and ferroptosis in vitro. Results: Our prediction model, comprising seven genes (AHNAK2, ARNTL2, CD27, LTB, SLC15A1, SLC2A1, and SYT1), has shown potential in predicting the prognosis of individuals diagnosed with LUAD. Notably, AHNAK2 impedes ferroptosis, promoting LUAD progression in vitro. Conclusions: Our research suggests that ferritinophagy-associated genes are promising prognostic markers for LUAD and lay the groundwork for further exploration of ferritinophagy’s role in LUAD. Furthermore, we present AHNAK2 as a novel regulator of ferroptosis, which requires further investigation to understand its mechanism.

## 1. Introduction

Lung cancer represents a significant threat to global health, with over two million new cases annually. NSCLC is the most common type, constituting over 80% of lung cancer cases. LUAD, a subtype of NSCLC, represents more than 40% of all cases [1,2]. Various factors contribute to LUAD development. Despite recent advances in diagnosis and treatment, survival rates remain low, resulting in poor prognosis. Therefore, it is critical to conduct comprehensive studies to unravel the molecular mechanisms of LUAD and identify new molecular markers that could enhance patient outcomes. Such research could deepen our understanding of the disease and lead to more effective prevention and treatment strategies.

Ferritinophagy leads to an overload of Fe^2+^ and reactive oxygen species (ROS), which triggers ferroptosis and subsequent cell death [3]. Recent studies have uncovered dysregulation in genes related to ferritinophagy in cancer, highlighting their potential as targets for cancer therapy [4,5]. Ferroptosis, a type of controlled cell death identified recently, is characterized by iron accumulation and irreversible lipid peroxidation, leading to cell demis [6,7]. The gene NCOA4, associated with ferritinophagy, enhances ferritin degradation and promotes ferroptosis [8]. Another ferritinophagy-related gene, FTH1, has been shown to inhibit ferroptosis in Parkinson’s disease [9]. Research on ferritinophagy in LUAD, however, is limited.

The AHNAK2 gene is highly expressed in various cancer tissues [10] and promotes LUAD progression through multiple signaling pathways [11,12,13]. In NSCLC, mutations in AHNAK2 are associated with a more active immune microenvironment [14]. However, the role of AHNAK2 in ferritinophagy and ferroptosis in LUAD is still unclear.

This study aims to develop a predictive model for individuals diagnosed with LUAD using data from The Cancer Genome Atlas (TCGA). We constructed the model by selecting seven genes linked to ferritinophagy and assessed its accuracy using three gene expression datasets from the Gene Expression Omnibus (GEO). We also explored the relationships among different aspects of ferritinophagy, immune cell infiltration, tumor mutation burden (TMB), microsatellite instability, immune evasion, and drug sensitivity. We validated the expression of the model genes (AHNAK2, ARNTL2, CD27, LTB, SLC15A1, SLC2A1, and SYT1) using an scRNA-seq dataset and analyzed their distribution across various cell subtypes. Additionally, we conducted knock-down experiments on the AHNAK2 gene in A549 and H1299 cells to examine its effects on proliferation, migration, invasion, and ferroptosis.

Our results indicate that genes associated with ferritinophagy could serve as valuable prognostic markers for LUAD and support further research into ferritinophagy’s role in this condition.

## 2. Materials and Methods

### 2.1. Data Source

Expression data and clinical information were acquired from three datasets—GSE31210 [15], GSE68465 [16], and GSE72094 [17]—which were retrieved from GEO database using the Bioconductor R package “GEOquery” [18]. For the analysis of this study, samples with comprehensive clinical information were chosen. Gene expression data of LUAD patients were obtained from the TCGA using the R package “TCGAbiolinks” [19] (R4.2.0), including 26 normal tissues and 187 tumor tissues with complete clinical information. In addition, somatic mutation data of 140 LUAD patients were acquired from the “Masked Somatic Mutation” category in TCGA. The ferritinophagy-related genes were retrieved from GeneCards website (https://www.genecards.org/, accessed on 10 December 2023) using the keyword “ferritinophagy”, yielding a total of 15 genes (NCOA4, USP24, FTH1, FTL, SNCA, HERC2, BECN1, ELAVL1, TNF, FBXW7, ATG5, ATG16L1, ATG7, ZFP36, and CYB561A3). GSE131907 [20] was downloaded from GEO database, the species is Homo sapiens, and the detection platform is GPL16791 Illumina HiSeq 2500. Samples were extracted from LUAD tissues and adjacent tissues. Eleven LUAD tissues samples were included in this study.

### 2.2. Analysis of Ferritinophagy-Related Genes

In the TCGA, the investigation employed the R package “gpubr” to evaluate the variation in gene expression associated with ferritinophagy in normal and tumor samples. The “maftools” [21] package was utilized to aggregate and visualize the mutation data of ferritinophagy-related genes. The “RCircos” [22] package was performed to visualize the genomic locations of ferritinophagy-related genes on chromosomes, using chromosomal data of the R package and gene location information from the GENCODE [23] database. The expression levels of genes related to ferritinophagy were extracted and analyzed for correlations using Pearson’s analysis. The outcomes were displayed utilizing the “corrplot” package.

### 2.3. Subtype Analysis

In the TCGA dataset, scores from single sample gene set enrichment analysis (ssGSEA) were calculated by implementing the “gsva” function from the “GSVA” [24] package. Based on the median score, patients were categorized into high and low ferritinophagy score groups. For purpose of describing the gene association patterns among the different patients, the study employed the Weighted Gene Correlation Network Analysis (WGCNA) [25] approach. The method has been executed to detect sets of genes with collaborative alterations and to identify genes that hold the potential to be used as biomarkers or targets for therapy. The analysis was conducted, ensuring a minimum of 50 genes in each module. The parameters used included a soft power of 10, a combined shear height of 0.25, and a minimum distance of 0.2. Gene-network modules were identified in different scoring groups.

The objective of this section was to cluster gene expression data utilizing the Consensus Clustering technique in order to discern unique subtypes of LUAD. The microarray gene expression data were analyzed using the package “ConsensusClusterPlus” [26]. The number of clusters was systematically varied between 2 and 10, with 100 replicates performed on 80% of the total samples, clusterAlg = “km”, distance = “euclidean”. The distribution among subtypes was shown using the “PCAtools” package. The DESeq2 [27] package was utilized for analyzing the mRNA expression differences between subtypes. A threshold of mRNAs differential expression between subtypes was defined as “adjusted *p* < 0.05 and |log2FC| > 1”.

### 2.4. Model Construction and Evaluation

In the process of constructing diagnostic models, the least absolute shrinkage and selection operator (LASSO) algorithm is frequently employed due to its ability to alleviate over-fitting through regularization and enhance model accuracy. Through Cox regression analysis on ferritinophagy-related genes, genes with prognostic significance were identified. These genes were then further scrutinized using the “glmnet” package. The model formulation was as follows:

$$risk\;sore=h_{0}\left(t\right)\exp\left(\Sigma_{j=1}^{n}c_{0}\mathrm{e}f_{j}\times x_{j}\right)$$

Based on the utilization of median prognostic risk scores, the samples were categorized into high-risk and low-risk groups. The “ggrisk” package was utilized to generate a map of risk factors, while the “survminer” was employed to visualize the curves. The model’s precision was measured by computing the area under the curve (AUC) for time points at 1, 3, and 5 years utilizing the “timeROC” [28] R package. Cox regression analysis was performed to assess the independence of the risk scores from other clinical factors. The “rms” package was used to create a nomogram and calibration curves.

### 2.5. Functional Enrichment Analysis

The differential expression analysis was performed using package “Deseq2”. To be considered as differential genes, the fold change must have an absolute value higher than 1 and the adjusted *p*-value should be lower than 0.05. The R package “ClusterProfiler 4.12.6” [29] was used to perform gene ontology (GO) [30] annotation and pathway enrichment analysis using the Kyoto Encyclopedia of Genes and Genomes (KEGG) [31]. If the FDR *p* value was below 0.05, the statistical significance of the results was acknowledged.

Gene set enrichment analysis (GSEA) was applied to the gene expression data acquired from TCGA. The gene collection “c2.cp.kegg.v7.1.entrez.gmt” was sourced from the Molecular Signatures Database (MSigDB) [32] and used in the GSEA analysis. A pathway was deemed significantly enriched if the *p*-value adjustment fell below 0.05.

A protein–protein interaction network of differentially expressed genes between high-risk and low-risk groups was constructed using the STRING database, with a correlation coefficient of 0.95. Export PPI results from STRING database and visualize them using Cytoscape 3.8.0. In addition, the MOCODE plugin was used to analyze the hub genes in the PPI network. Next, we will use the GOSemSim package 2.31.2 to analyze the importance of hub genes.

### 2.6. Immune-Related Analysis

Package “IOBR” [33] integrated several published methods for quantifying tumor microenvironment (TME), such as CIBERSORT [34], TIMER [35], xCell [36], MCPcounter [37], ESITMATE [38], EPIC [39], quantTIseq [40], etc. The immune cell infiltration was analyzed using this package. Next, we obtained the expression values of immune checkpoint genes in both high and low risk groups.

### 2.7. Somatic Mutation, MSI and TIDE Analysis

We employed the “Maftools” package for the examination of somatic mutations and TMB. We download MSI data of LUAD from UCSC (https://xena.ucsc.edu/, accessed on 24 December 2023). By inputting the expression data into the Tumor Immunity Dysfunction and Exclusion (TIDE) [41] website (http://tide.dfci.harvard.edu/, accessed on 24 December 2023), we obtained the TIDE score, dysfunction score, and exclusion score.

### 2.8. Analysis of Drug Sensitivity

The Genomics of Drug Sensitivity in Cancer (GDSC) database offers a comprehensive platform to investigate the response of tumors to drugs and identify sensitive genomic markers. The “pRRophetic” [42] algorithm was utilized to build a ridge regression model utilizing the expression profile of cell lines acquired from the Cancer Cell Line Encyclopedia (CCLE). This model was employed to anticipate the sensitivity towards widely used anti-cancer medications, evaluated using the IC50 value.

### 2.9. Single Cell Sequencing Analysis

We create the expression matrix of GSE131907 as a Seurat object. Cells with mitochondrial gene content > 20% and features < 200 or >10,000 were filtered. We use “LogNormalize” method to standardize the data. Two thousand hypervariable genes were returned for downstream dimension reduction analysis. Next, we proceeded to employ principal component analysis (PCA) in order to identify the dominant principal component (PC). Subsequently, the *p*-value distribution was visualized using the Elbowplot function. We set the value of resolution to 1.2 to cluster and optimize the data. We utilized the “RunUMAP” function to reduce dimensionality and enable visualization and exploration of datasets, selecting the top 15 PCs for uniform manifold approximation and projection (UMAP) analysis. Human primary cell atlas data was used to automatically annotate cells. To analyze the dynamic evolution of gene expression, the “Monocle 2.30.0” [43] package was used. Intercellular communication network was built through “CellChat 1.6.1” [44].

### 2.10. Cell Culture and Quantitative Real-Time PCR (RT-qPCR)

Normal lung epithelial cell HBE and three human LUAD cell lines (A549, H1299, and SPCA-1) were acquired from the cell bank of the Chinese Academy of Medical Sciences (Shanghai, China). The cells were cultured in RPMI 1640 medium from Corning, New York, USA and supplemented with 10% FBS. They were then incubated at 37 °C with 5% CO_2_. Trizol reagent (Life Technologies, Carlsbad, CA, USA) was utilized for the extraction of total RNA, which was then subjected to reverse transcription and RT-qPCR analysis using a reverse transcription kit and SYBR (GenePharma, Shanghai, China). Primer sequences for quantitative RT-PCR and siRNAs of AHNAK2 (GenePharma, Shanghai, China) were listed in Table S1.

### 2.11. CCK8, Colony Formation, Transwell, and Wound-Healing Assay

Cell viability and proliferation were measured using a commercial CCK-8 solution (Beyotime, Guangzhou, China). LUAD cells were seeded in 96-well plates with a density of 4 × 10^3^ cells per well. Cells were treated with 10 μL of CCK-8 reagent for 2 h at time intervals of 24, 48, and 72 h, and the absorbance at 450 nm was measured to evaluate cell growth. To measure the effect of erastin (Beyotime, Guangzhou, China) on the viability of LUAD cells, cells were treated with erastin (10 μM) or DMSO for 24 h.

LUAD cells (1 × 10^3^ cells/well) with indicated transfection were seeded and grown into 6-well plates for a duration of two weeks. The medium for cultivating cells was replaced every three days. Next, the colonies produced were rinsed, immobilized, and dyed with crystal violet (Beyotime, Guangzhou, China).

LUAD cells were deprived of serum in RPMI 1640 medium for a duration of 8 h. LUAD cells (2 × 10^5^ cells) were inoculated into the superior cavity with pre-embedded matrigel (Corning, New York, NY, USA) and cultured for 18–24 h to invade the inferior cavity. After immobilization, the cells were stained with crystal violet and observed using a microscope.

LUAD cells were inoculated in 48-well plates with a cell density of 2 × 10^5^ cells per well and incubated for a duration of 12 h. Following the fusion of the cells, the cell scratches were made with the 100 μL pipet tip. Cultivation of the cells continued, and photos were taken to calculate the migration distance after 24 h.

### 2.12. Fe^2+^, ROS, GSH, and MDA Assay

A Cell Ferrous Iron Colorimetric Assay Kit (Elabscience, Wuhan, China) was used to detect the levels of Fe^2+^, while the Reactive oxygen species Assay Kit (biosharp, Hefei, China) was used to measure ROS. The Total glutathione/Oxidized glutathione assay kit (Jiancheng, Nanjing, China) was employed to determine the levels of GSH, and the Malondialdehyde (MDA) assay kit (Jiancheng, Nanjing, China) was utilized to assess MDA levels. All of the aforementioned tests were conducted in accordance with the experimental protocol given by the supplier of the reagent. All cells were pretreated with media containing erastin (10 μM) to induce ferroptosis.

### 2.13. Transmission Electron Microscopy

Cells were fixed with 2.5% glutaraldehyde at room temperature for 1 h and then at 4 °C overnight. Next, the cells were soaked in 1% osmium tetroxide, washed with phosphate buffer, dehydrated with ethanol, and embedded in epoxy resin. Samples were cut with ultramicrotome and stained with uranyl acetate and lead citrate. The slices were observed with a transmission electron microscope.

### 2.14. Statistical Analysis

Statistical analysis was conducted utilizing R software version 4.0.2 and GraphPad Prism 8.0. To analyze continuous variables, we assessed normality and used an independent Student’s *t*-test for normally distributed variables. For non-normally distributed variables, we utilized the Wilcoxon rank-sum test. One-way analysis of variance (ANOVA) was used to compare the variance among different groups. The chi-square or Fisher’s exact test was employed to compare the categorical variables. Survival analysis was performed using the R package “survival”, and the Kaplan–Meier survival curve was employed to visualize variations in survival. To assess the importance of the difference in survival time among the different groups, the log-rank test was utilized. Furthermore, the R package “survival” was utilized to perform Cox regression analysis (both univariate and multivariate), and the R package “glmnet” was employed for LASSO analysis. Statistical significance was determined by considering *p*-values less than 0.05 in two-tailed tests.

## 3. Results

### 3.1. Landscape of Ferritinophagy-Related Genes in LUAD

**Figure 1:**
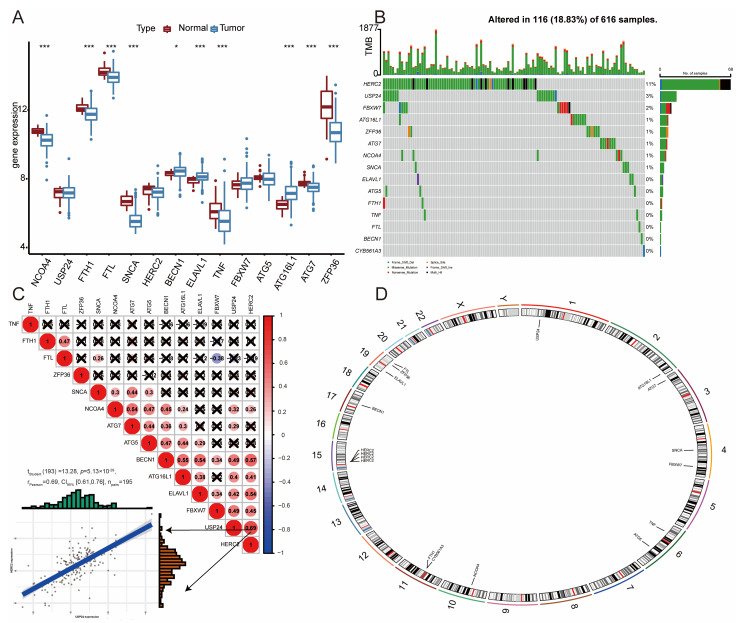
Landscape of ferritinophagy-related genes in LUAD. (**A**): Box plot showing the difference in ferritinophagy-related genes between normal tissue and tumor tissue. (**B**): Mutation plot of ferritinophagy-related genes. (**C**): Heatmap showing the correlation of ferritinophagy-related genes. Blue represents negative correlation, red represents positive correlation. (**D**): Chromosomal location map of ferritinophagy-related genes. * *p* < 0.05; *** *p* < 0.001.

The “sva” package was utilized to mitigate batch effects across datasets (Figure S1A,B). The baseline characteristics of the four datasets are detailed in Table S1. Among the fourteen ferritinophagy-related genes studied, ten exhibited significant differences between tumor and normal samples: NCOA4, FTH1, FTL, SNCA (all *p* < 0.0001), BECN1 (*p* = 0.031), ELAVL1 (*p* = 0.00023), TNF (*p* = 0.00074), ATG16L1, ATG7, and ZFP36 (all *p* < 0.0001). BECN1, ELAVL1, and ATG16L1 were highly expressed in tumor tissues, while NCOA4, FTH1, FTL, SNCA, TNF, ATG7, and ZFP36 showed low expression levels (Figure 1A). Mutation analysis revealed that HERC2 and USP24 were the most mutated genes, in that order (Figure 1B). Correlation analysis indicated a positive correlation among most ferritinophagy-related genes, with HERC2 and USP24 showing the strongest positive correlation (0.69) (Figure 1C). Chromosomal locations of these genes are depicted in Figure 1D, underscoring the significant association of ferritinophagy with LUAD progression.

### 3.2. WGCNA

**Figure 2:**
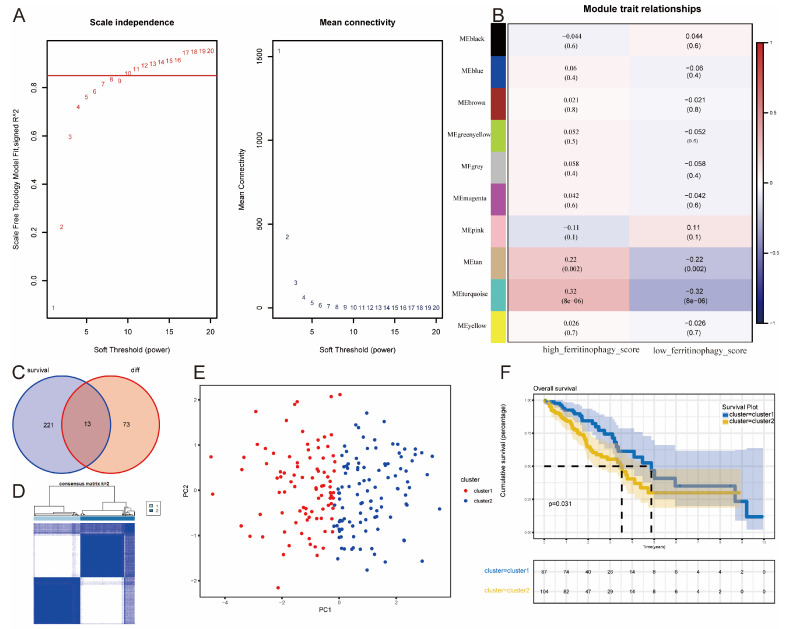
WGCNA of high and low ferritinophagy scoring groups on the TCGA expression matrix. (**A**): Scale-free index and mean connectivity in WGCNA. (**B**): Correlation heatmap between ferritinophagy scores and gene modules. (**C**): Venn diagram displaying the intersection of differentially expressed genes and survival-related genes. (**D**): Sample clustering plot with K = 2. (**E**): PCA showing the distribution of subtypes. (**F**): Survival analysis (KM method) of two subtypes.

Using ssGSEA, ferritinophagy scores were calculated for each sample in the TCGA dataset. To investigate the expression patterns of ferritinophagy-related genes in LUAD, we conducted a weighted gene co-expression network analysis (WGCNA) on the TCGA expression matrix. Applying a soft threshold of 10, we achieved a scale-free network and identified 10 modules (Figure 2A). The MEturquoise module, comprising 1987 genes, was the most strongly associated with ferritinophagy scores (Figure 2B). Further analysis of the MEturquoise module through differential and survival analyses identified 13 intersecting hub genes (ADCY9, B3GALNT1, B4GALT4, CCT3, DARS2, DPY19L1, DSG2, FAT1, INTS8, LPGAT1, PHKA1, RALGPS2, SRPK1), as shown in Figure 2C. Cluster analysis using the R package “ConsensusClusterPlus” based on the expression of these 13 genes differentiated patients into Cluster 1 and Cluster 2 (Figure 2D), effectively distinguishing between two LUAD subtypes (Figure 2E). Survival analysis showed that individuals in Cluster 2 had poorer prognoses compared to those in Cluster 1 (Figure 2F).

### 3.3. Construction of Prognostic Model

**Figure 3:**
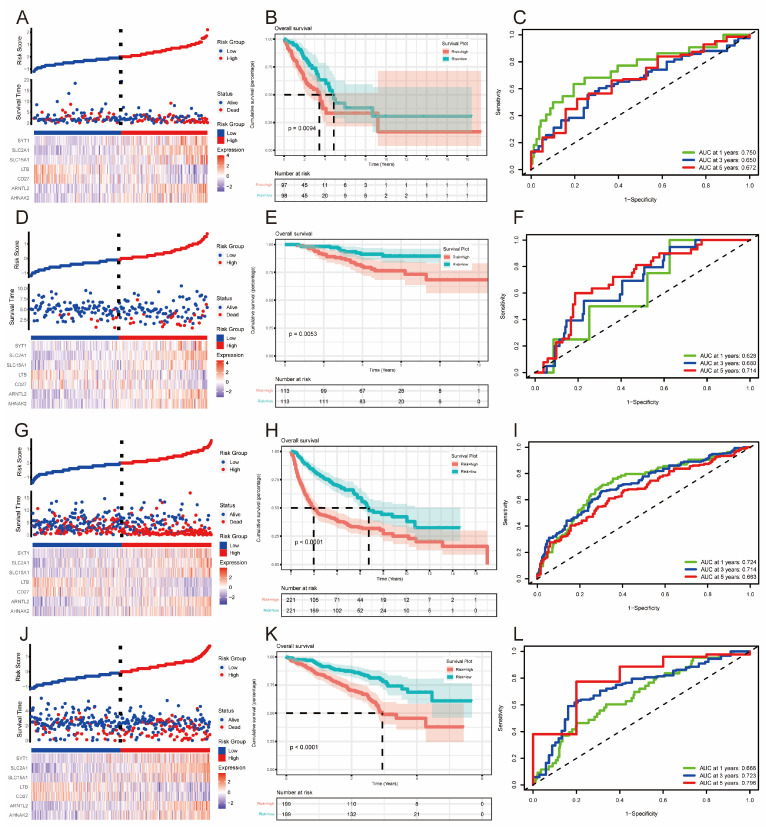
Curve scatter plots, cumulative scatter plots, KM curves and time-dependent ROC curves: TCGA (**A**–**C**), GSE31210 (**D**–**F**), GSE68465 (**G**–**I**), and GSE72094 (**J**–**L**).

We conducted a differential gene analysis to identify distinctions between two ferritinophagy-related subtypes, uncovering 418 differential genes. A univariate Cox regression analysis of these genes identified 28 with significant prognostic value. We then applied LASSO regression to refine these findings, selecting the optimal number of genes based on the optimal λ value (Figure S2A,B). Subsequently, a Cox regression model incorporating seven genes was established. Risk scores were calculated for both the training (TCGA) and verification (GEO) sets. Patients were categorized into two groups based on the median risk score. The risk factor chart for the training dataset showed a higher mortality rate in the high-risk group (Figure 3A). Survival analysis confirmed a poorer prognosis for the high-risk group (*p* = 0.0094, as depicted in Figure 3B). Additionally, ROC analysis revealed AUC values of 0.750, 0.650, and 0.672 for 1, 3, and 5 years, respectively, in the training set (Figure 3C). These results were validated across three GEO datasets (Figure 3D–L).

### 3.4. Biological Functional Analysis

**Figure 4:**
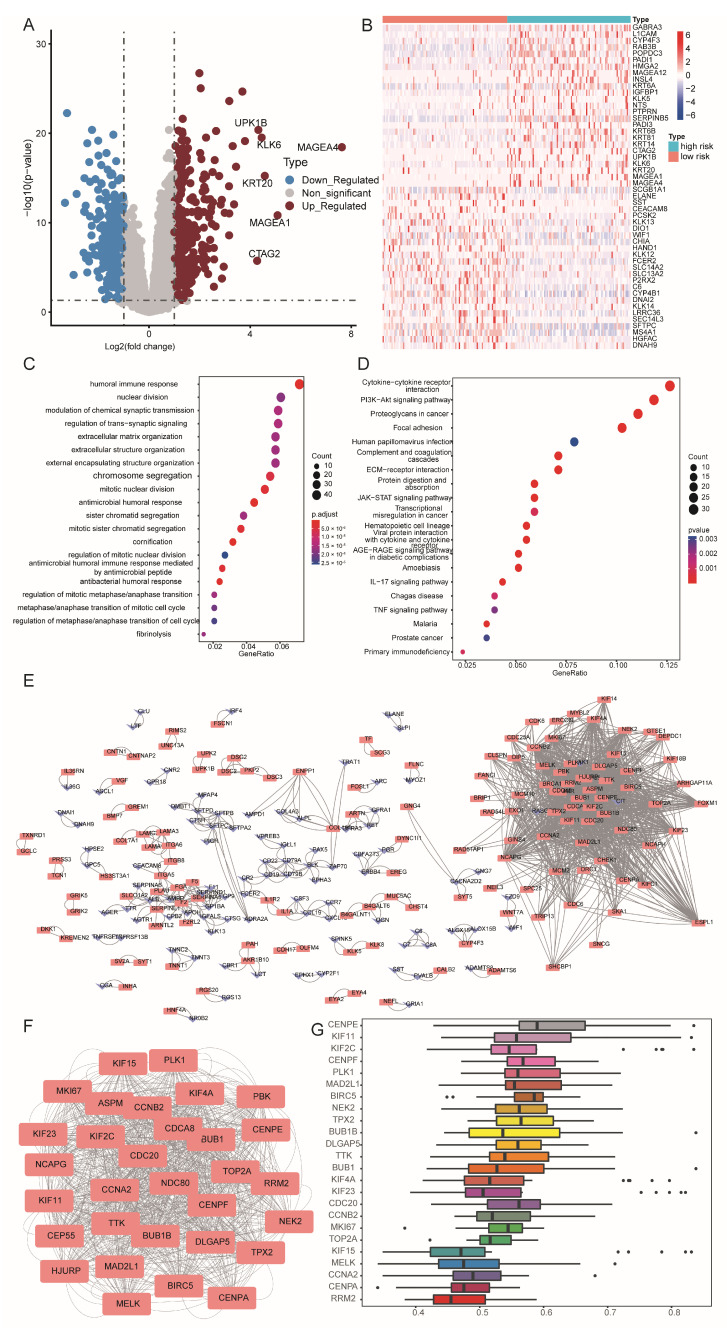
Biological function analysis of 647 differentially regulated genes between the high and low-risk groups. (**A**): Volcano plot showing differentially expressed genes. (**B**): A heatmap displaying the top 100 differentially expressed genes. (**C**): GO functional enrichment analysis. (**D**): KEGG functional enrichment analysis. (**E**): PPI network. Blue represents low expression, while pink represents high expression. (**F**): Hub gene module. (**G**): Friends analysis.

**Figure 5:**
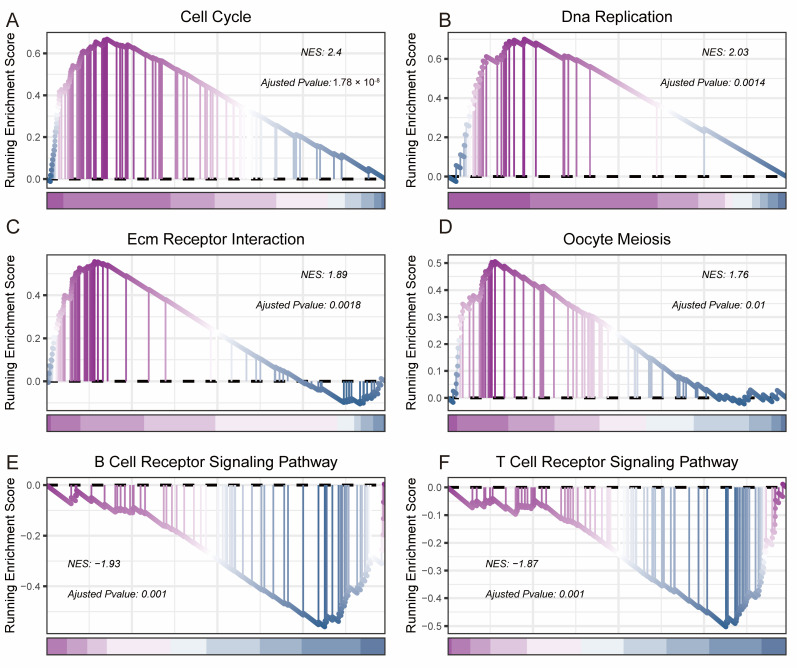
GSEA analysis of 647 differentially regulated genes between the high and low-risk groups. Enriched in cell cycle (**A**), DNA replication (**B**), ECM receptor interaction (**C**), oocyte meiosis (**D**), B cell receptor signaling pathway (**E**), and T cell receptor signaling pathway (**F**).

To elucidate the biological relevance of our risk model, we compared gene expression between the high and low-risk groups. Analysis showed 647 differentially regulated genes, with 298 down-regulated and 349 up-regulated (Figure 4A). A heatmap displays the 100 most significantly altered genes (Figure 4B). GO analysis on these genes highlighted enriched categories in immune response, signal transmission regulation, and cell cycle control (Figure 4C). KEGG pathway analysis indicated that many differentially expressed genes participate in several signaling pathways related to tumor development (Figure 4D). We proceeded to perform PPI network analysis on the differentially expressed genes and visualized them using Cytoscape (Figure 4E), where red represents high expression and blue represents low expression. Then, we used the MCODE plugin to display the hub genes, with a score of 28.533 for cluster1 (Figure 4F), resulting in 24 hub genes. Friends analysis showed that CENPE may be the most important gene, followed by KIF11 (Figure 4G). GSEA revealed that the high-risk group exhibited significant enrichment in pathways related to the cell cycle (Figure 5A), DNA replication (Figure 5B), interaction with ECM receptors (Figure 5C), and oocyte meiosis (Figure 5D). Conversely, the low-risk group showed enhanced pathways associated with B-cell and T-cell receptor signaling (Figure 5E,F). This analysis revealed complex differences in biological functions between the groups, especially in tumor cell cycle regulation and immune signal transmission.

### 3.5. Immune-Related Analysis

**Figure 6:**
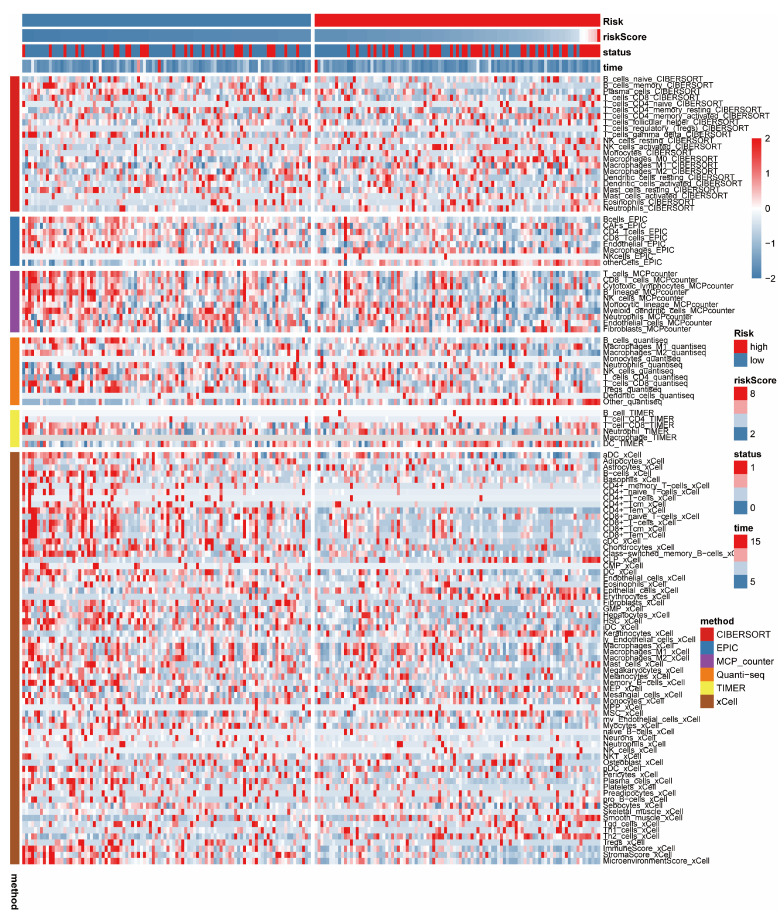
Immune analysis. A heatmap displays enrichment scores of six immune infiltration analyses.

Using six widely recognized methods for immune infiltration analysis (CIBERSORT, EPIC, MCP Counter, Quanti-seq, TIMER, and xCell), we examined immune cell presence. The results are displayed in a heatmap (Figure 6) illustrating the immune infiltration scores from these methods. Our analysis revealed distinct differences in immune cell scores between two groups, with more detailed results available in Supplementary Figures (Figure S3A–F). The group with the lowest risk showed higher immune, stromal, and ESTIMATE scores, indicating a more favorable prognosis (Figure S3G). Additionally, we studied the expression of 14 immune checkpoint genes, finding significant expression differences in five of these genes (CD244, CD96, CTLA4, TGFβr1, VTCN1), as shown by differential analysis (Figure S3H). The low-risk group demonstrated higher immune infiltration scores, suggesting a stronger immune response and a better prognosis.

### 3.6. Multi-Omics Analysis

**Figure 7:**
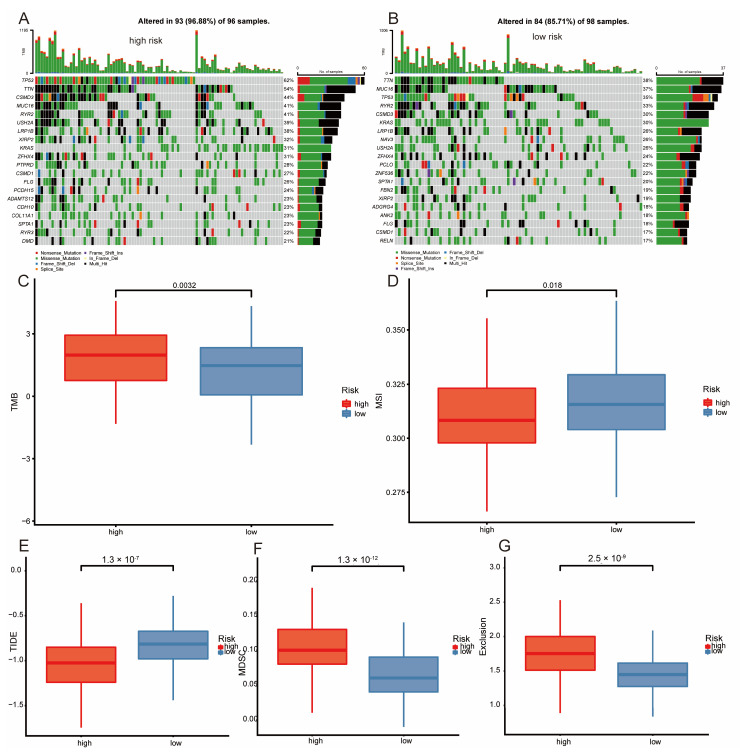
Multi-omics analysis of high and low risk groups. (**A**,**B**): Mutation landscapes. Box plot showing the difference in tumor mutation burden (**C**), microsatellite instability (**D**), TIDE (**E**), MDSC (**F**), and exclusion (**G**) between high and low risk groups.

Mutation analysis indicated that the high-risk group had a higher frequency of gene mutations (96.88% vs. 85.71%, Figure 7A,B), particularly in the TP53 gene, where mutations occurred more frequently (62% vs. 35%). Mutations in the TP53 gene often imply more aggressive tumors with poorer prognosis [45]. Mutation of TP53 gene leads to the decrease of autophagy level of cancer cells, which is not easy to cause ferroptosis and is beneficial to tumor growth [46]. Analysis of tumor mutational burden revealed that the TMB exhibited a considerably greater magnitude in the high-risk category (Figure 7C). The high-risk group exhibited lower MSI score (Figure 7D) and TIDE value (Figure 7E). Additionally, the high-risk group displayed significantly higher levels of MDSC (Figure 7F) and exclusion (Figure 7G). The findings indicated that individuals in the high-risk category may exhibit a more favorable reaction to immunotherapy.

### 3.7. Analysis of Drug Sensitivity

**Figure 8:**
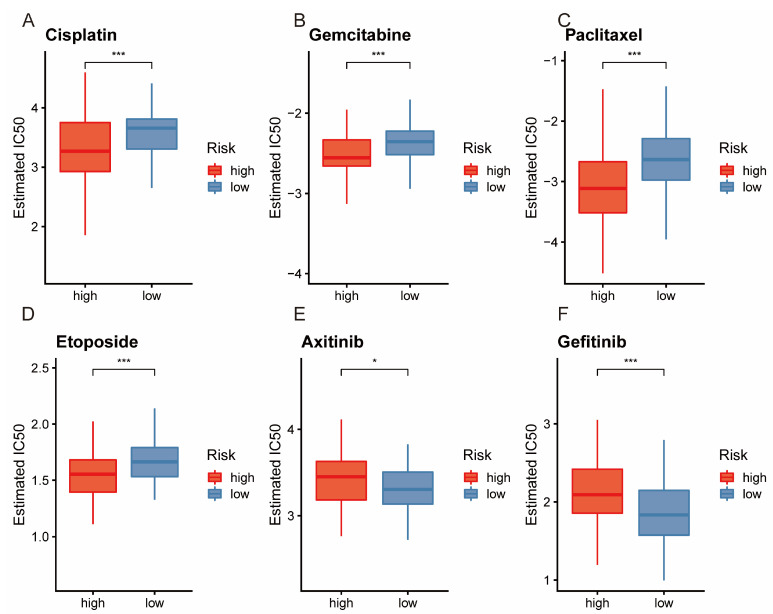
Drug sensitivity analysis. Cisplatin (**A**), gemcitabine (**B**), paclitaxel (**C**), etoposide (**D**), axitinib (**E**), and gefitinib (**F**). * *p* < 0.05; *** *p* < 0.001.

Significant differences in drug sensitivity were observed among various agents. The high-risk group was more responsive to Cisplatin (Figure 8A), Gemcitabine (Figure 8B), Paclitaxel (Figure 8C), and Etoposide (Figure 8D). Conversely, the low-risk group showed higher sensitivity to Axitinib (Figure 8E) and Gefitinib (Figure 8F). These findings can guide the selection of more suitable and effective treatments for different patient groups.

### 3.8. Independent Prognosis Analysis and Constructing the Nomogram and Calibration Curves

We integrated clinical information such as patient gender, age, and tumor grade to assess whether the prognostic risk signature was independent of clinical variables. In the TCGA dataset, only stage and ferritinophagy risk score emerged as independent prognostic factors (Figure S4A,B). Similarly, in the GSE31210 dataset, stage and risk score proved statistically significant (Figure S4C,D). In the GSE68465 dataset, age, nodal involvement (N), tumor size (T), and ferritinophagy risk score were significant (Figure S4E,F). In the GSE72094 dataset, stage, gender, and risk scores were identified as independent factors (Figure S4G,H). We developed a nomogram and calibration curves using clinical information and risk scores to predict overall survival (OS) at 1, 3, and 5 years for the TCGA cohort (Figure S4I,J). This model’s predictive capability was confirmed through validation in three GEO cohorts (Figure S4K–P). Due to limited survival data beyond 5 years for LUAD patients, the nomogram and calibration curves were presented only for 1, 2, and 3 years in GSE72094.

### 3.9. Validation of Model Genes with Single-Cell Sequencing

**Figure 9:**
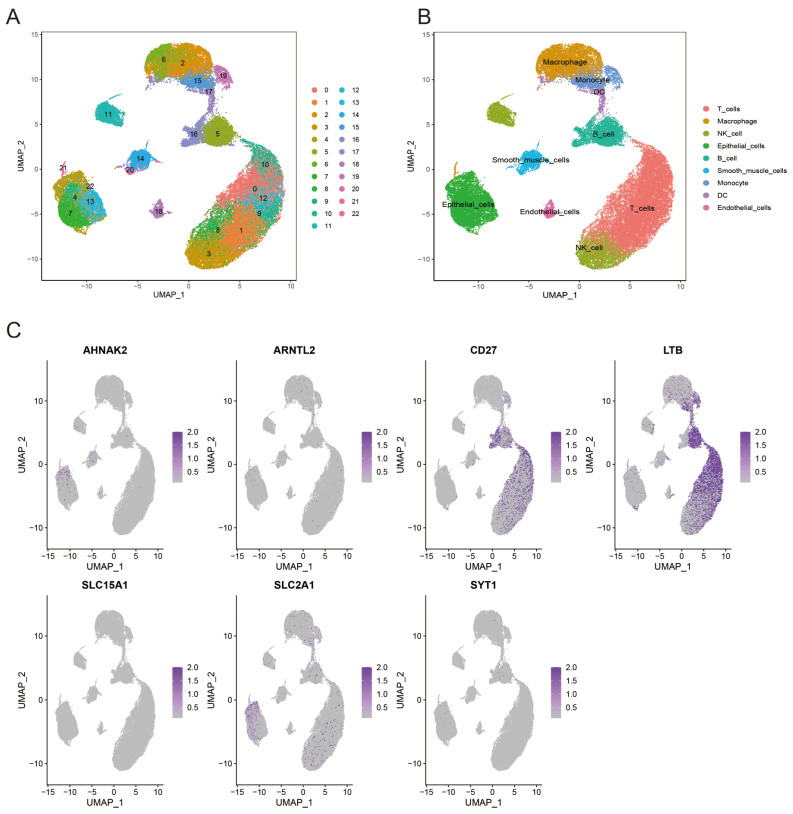
Single-cell RNA-sequencing analysis of seven prognosis genes. (**A**) UMAP plot colored by various clusters. (**B**) UMAP plot of the cell types. (**C**) Distribution of prognostic genes.

After quality control and normalization of scRNA-seq data, we analyzed 42,529 cells, dividing them into 22 clusters based on the top 1500 variable genes (Figure 9A). These clusters were annotated as 9 cell types (Figure 9B). We then examined the expression of model genes (AHNAK2, ARNTL2, CD27, LTB, SLC15A1, SLC2A1, SYT1) at the cellular level (Figure 9C). AHNAK2 and SLC2A1 were primarily expressed in epithelial cells, while CD27 and LTB were predominantly associated with T cells, potentially influencing tumor immunity.

### 3.10. Identification of AHNAK2^+^ Epithelial Cells Subtype

**Figure 10:**
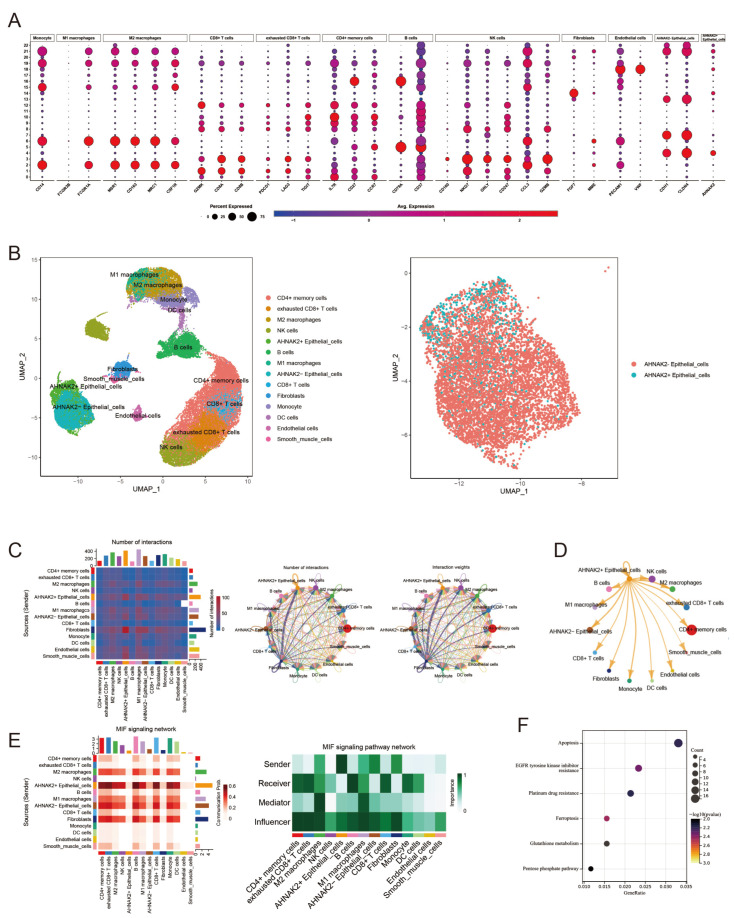
Identification of AHNAK2^+^ epithelial cells subtype. (**A**): Marker genes for cell annotation. (**B**): UMAP plot of the cell types. (**C**): Interaction number and weight plot of LUAD cells. (**D**): Cell–cell communications between AHNAK2^+^ epithelial cells with other cells. (**E**): The heatmaps show the communication probability and signal role (Sender, Receiver, Mediator, and Influencer) that the cell types play in the MIF signaling pathway. (**F**): KEGG analysis of differential expression genes between AHNAK2^+^ and AHNAK2^−^ epithelial cells.

From earlier analysis, AHNAK2 was primarily found in epithelial cells. We re-annotated these cells, distinguishing AHNAK2^+^ epithelial cells (cluster 4) from AHNAK2^−^ cells (clusters 7, 13, and 22). Clusters were re-annotated based on cell markers (Figure 10A), with results displayed in Figure 10B. Cell–cell communication analysis revealed that AHNAK2^+^ epithelial cells exchanged more signals than their AHNAK2^−^ counterparts (Figure 10C). Further investigation showed that AHNAK2^+^ epithelial cells primarily interacted with M1 and M2 macrophages, and exhausted CD8^+^ T cells, potentially leading to an immunosuppressive environment conducive to LUAD progression (Figure 10D). Ligand-receptor interaction analysis indicated that the MIF signaling pathway was pivotal in communication between AHNAK2^+^ epithelial cells and other cells (Figure S5), with AHNAK2^+^ cells being key signal senders and various immune cells including macrophages, T cells, and B cells as main recipients (Figure 10E). Lastly, KEGG analysis of genes differentially expressed between AHNAK2^+^ and AHNAK2^−^ epithelial cells highlighted pathways, including those related to EGFR tyrosine kinase inhibitors and platinum resistance, particularly emphasizing ferroptosis and glutathione metabolism pathways (Figure 10F).

### 3.11. AHNAK2 Was Significantly Up-Regulated in LUAD Tissues and Cells

**Figure 11:**
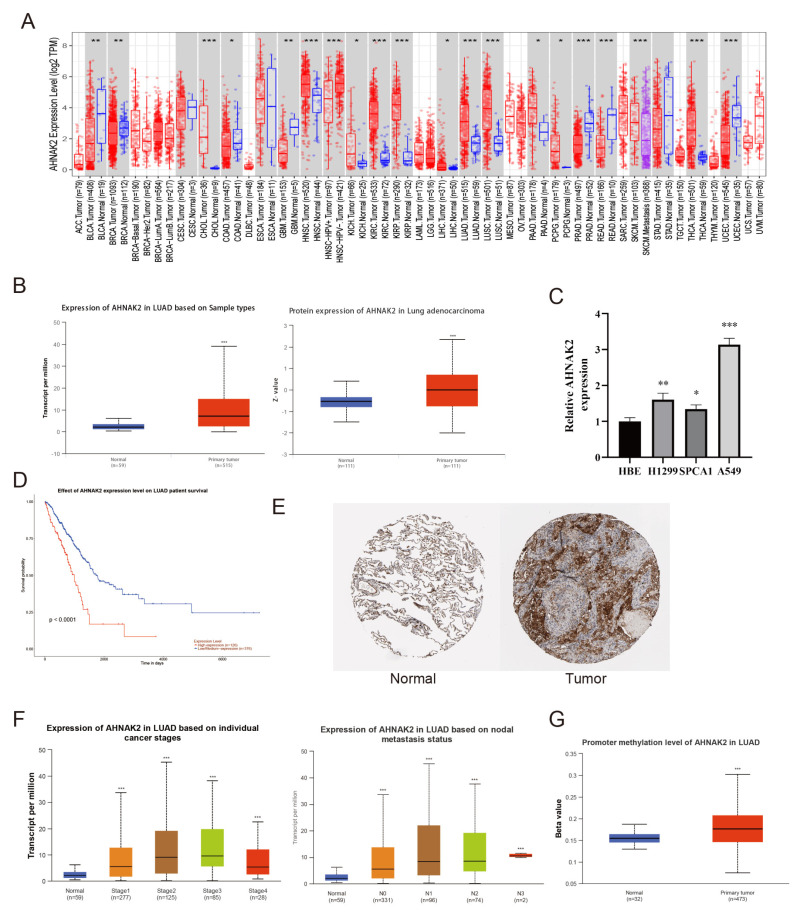
AHNAK2 was highly expressed in LUAD tissues and cells. (**A**): Expression of AHNAK2 between tumor and normal tissue. (**B**): mRNA and protein expression of AHNAK2 in TCGA and CPTAC. (**C**): RT-qPCR analysis of AHNAK2 mRNA expression in four cell lines. (**D**): The overall survival of patients. (**E**): Immunohistochemistry for protein expression of AHNAK2 in HPA. (**F**): Expression of AHNAK2 based on individual cancer stages and nodal metastasis status. (**G**): Promoter methylation level of AHNAK2 in LUAD. * *p* < 0.05; ** *p* < 0.01;*** *p* < 0.001.

Pan-cancer analysis revealed significant upregulation of AHNAK2 across most tumor tissues (Figure 11A). Data from the UALCAN database showed that both AHNAK2 mRNA and protein levels were considerably higher in LUAD tissues compared to normal lung tissues (Figure 11B). Additionally, three LUAD cell lines showed increased AHNAK2 expression relative to human bronchial epithelial (HBE) cells (Figure 11C). In TCGA, patients with elevated AHNAK2 levels had a poorer prognosis (Figure 11D). Immunohistochemistry (IHC) results from the HPA databases showed higher AHNAK2 expression in LUAD tissues (Figure 11E). We also explored the relationship between AHNAK2 expression and tumor stage and lymph node metastasis in LUAD patients (Figure 11F), observing an increase in AHNAK2 expression with advancing tumor stages, followed by a decrease. Single-cell sequencing data showed a similar trend in AHNAK2 expression over tumor evolution (Figure S6). The reasons for this pattern are unclear, but it may involve a negative feedback mechanism regulating AHNAK2 expression. AHNAK2 methylation levels were higher in tumor tissues (Figure 11G).

### 3.12. Silencing AHNAK2 Suppresses the Cell Proliferation, Invasion, and Migration of LUAD Cells

**Figure 12:**
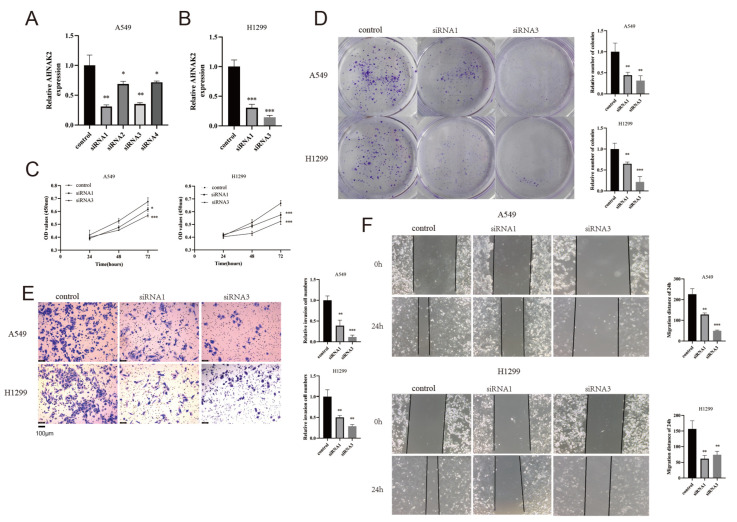
AHNAK2 promoted proliferation, invasion, and migration. (**A**,**B**): Expression of AHNAK2 after siRNAs interference. (**C**): CCK8 assay analyzed the proliferation. (**D**): The colony formation of LUAD cells. (**E**): Transwell assays of A549 and H1229 cells for evaluating cancer cell invasion. (**F**): Wound-healing assay of A549 and H1229 cells. * *p* < 0.05; ** *p* < 0.01;*** *p* < 0.001.

Four siRNA constructs targeting AHNAK2 were synthesized. We selected siRNA1 and siRNA3 for their superior silencing efficacy (Figure 12A,B). CCK-8 and colony formation assays indicated that silencing AHNAK2 impeded LUAD cell growth (Figure 12C,D). Additionally, AHNAK2 interference reduced the invasion capabilities of LUAD cells (Figure 12E). A wound-healing assay demonstrated that knocking down AHNAK2 decreased the migration of LUAD cells (Figure 12F).

### 3.13. AHNAK2 Attenuates Ferroptosis of LUAD Cells

**Figure 13:**
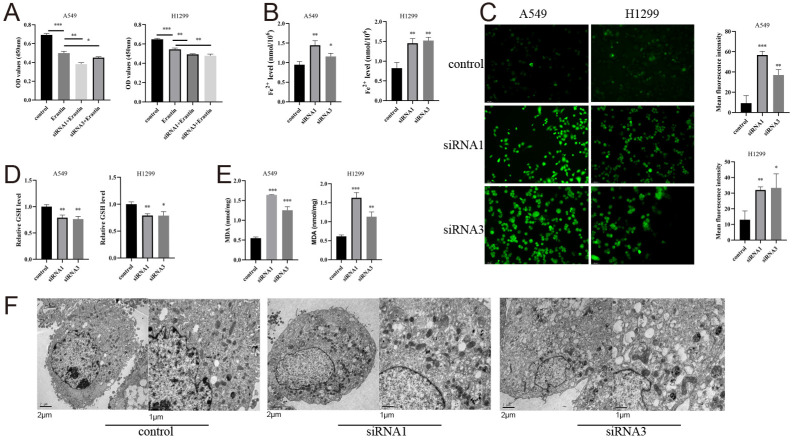
Silenced AHNAK2 inhibits the ferroptosis of LUAD cells. (**A**): After treatment with DMSO (control) and erastin (10 μM, others), CCK8 assay was evaluated at time of 72 h. Relative Fe^2+^ level (**B**), ROS level (**C**), GSH content (**D**), and MDA content (**E**) was evaluated in A549 and H1299 cells. All cells were treated with media containing erastin (10 μM) to induce ferroptosis. (**F**): Mitochondrial morphology observed by transmission electron microscopy. * *p* < 0.05; ** *p* < 0.01;*** *p* < 0.001.

Treatment with erastin diminished the viability of LUAD cells, and silencing AHNAK2 enhanced this effect (Figure 13A). AHNAK2 silencing also increased Fe^2+^ levels in LUAD cells (Figure 13B). ROS levels were assessed using fluorescence microscopy, showing that AHNAK2 reduced intracellular ROS (Figure 13C). GSH content decreased and MDA levels increased in AHNAK2-silenced cells (Figure 13D,E). Transmission electron microscopy revealed typical morphological changes indicative of increased ferroptosis in A549 cells following AHNAK2 downregulation (Figure 13F). These findings suggest that AHNAK2, a gene linked to ferritinophagy, influences the ferroptosis process in LUAD cells.

## 4. Discussion

Our study underscores the pivotal role of ferritinophagy in LUAD and highlights the necessity of identifying new biomarkers to target ferritinophagy in LUAD management. Despite the potential therapeutic benefits of ferritinophagy, its clinical application has been hindered by the lack of reliable markers. Thus, our research is critical for discovering potential biomarkers that could enhance the effectiveness of ferritinophagy-targeted cancer therapies.

In our research, we used data from TCGA, GEO, and scRNA-seq to categorize LUAD patients into two subtypes based on their ferritinophagy status. We also developed a prognostic model comprising seven ferritinophagy-related genes to evaluate their potential in predicting prognosis, immune infiltration, response to immunotherapy, and drug sensitivity. Moreover, we focused on the role of AHNAK2 in LUAD progression.

AHNAK2 is highly expressed in various cancer types, including LUAD [47], papillary thyroid cancer [48], and bladder cancer [49], and is associated with poor prognosis. AHNAK2 affects the migration and invasion of LUAD through the TGF-β/Smad3 pathway [13] and MAPK pathway [11]. In thyroid cancer, some scholars have shown that the NF-κB pathway [50], PI3K/AKT signaling pathway [51], and Wnt/β-catenin pathway [52] may partially explain the unfavorable prognosis in the high expression cohort of AHNAK2. AHNAK2 has also been shown to inhibit oxidative phosphorylation, a major source of ROS [47]. Based on previous findings and our current study, we suspect AHNAK2 plays a role in the ferroptosis process. The inhibition of ARNTL2 reduces the motility and invasive ability of PDAC cells in vitro and hinders tumor development in vivo [53]. ARNTL2 inhibits ferroptosis in LUAD and NSCLC [54,55]. CD27 has recently been identified as a highly promising target for immunotherapy [56,57]. CD27 is mainly expressed on CD4^+^ and CD8^+^ T cells [56], which is consistent with our analysis. Lymphotoxin beta (LTB) is a type II membrane protein of the TNF family and mainly expressed on B and T cells [58]. LTB is the ligand of lymphotoxin-β receptor (LTBR), and LTBR signaling pathway can enhance the effector function of T cells [59]. Rs4646227 of SLC15A1 has been identified as an independent indicator in bladder cancer [60], and SLC15A1 has been found to be a potential predictor of recurrence in LUAD [61]. On the other hand, SLC2A1 has been found to act as an oncogene that promotes cellular glycolytic metabolism [62]. SLC2A1′s involvement in METTL3-mediated glycolysis has been observed in colorectal cancer [63]. In addition, it is closely related to the ferritinophagy-related gene GPX4 [64]. The study found a correlation between SYT1 and response to radiation therapy for nasopharyngeal carcinoma [65]. SYT1 regulates neurotransmitter release by interacting with anionic phospholipids [66]. Additionally, SYT1 acts as a fast calcium ion sensor in Cryptobacterium hidradenum [67]. This study is the first to combine these seven genes to predict LUAD prognosis. We also discovered that the ferritinophagy-related gene AHNAK2 can inhibit ferroptosis in LUAD cells, although the specific mechanisms require further investigation.

This study has several limitations. Firstly, multicenter prospective clinical trials are necessary to validate the predictive effects of the relevant genes identified. Secondly, the absence of mechanistic experiments and related animal studies limits our ability to confirm the regulatory mechanisms of the model genes in LUAD. Additionally, although we have observed that AHNAK2 affects ferroptosis in LUAD cells, its specific biological mechanisms remain unclear; this will be an area of focus in future research. Addressing these limitations is essential to deepen our understanding of the disease’s underlying mechanisms.

## 5. Conclusions

We developed a prognostic model incorporating seven ferritinophagy-related genes using various bioinformatics methods for LUAD. We confirmed the expression of AHNAK2 and its impact on the proliferation, migration, invasion, and ferroptosis of LUAD cells in vitro. Our findings offer new potential biomarkers and therapeutic targets for LUAD.

## Data Availability

The datasets supporting the conclusions of this article are included within the article. Further inquiries can be directed to the corresponding authors.

## Supplementary Materials

The following supporting information can be downloaded at: https://www.mdpi.com/article/10.3390/bioengineering11111070/s1, Figure S1 Batch effect plot of the dataset. A: PCA representation of the dataset distribution before data correction. B: PCA representation of the dataset distribution after data correction; Figure S2 LASSO regression model for constructing prognostic gene signatures. A: Distribution of LASSO coefficients. B: The generated coefficient distribution plots; Figure S3 Immune analysis. Box plot showing the difference between the low and high risk groups using the CIBERSORT (A), EPIC (B), MCP_counter (C), Quanti-seq (D), xCell (E), and TIMER (F) algorithm. G: Stromal score, immune score, ESTIMATE score, and tumor purity of the low and high risk groups. H: Box plot showing the difference between the low and high risk groups in terms of immune checkpoint. * *p* < 0.05; ** *p* < 0.01; *** *p* < 0.001; Figure S4 Independent prognostic analysis of risk score. Forest plot showing the univariate and multivariate Cox regression analysis of risk score and clinical phenotype in the TCGA (A–B), GSE31210 (C–D), GSE68465 (E–F) and GSE72094 (G–H). Kaplan-Meier plot for predicting 1-year, 3-year, and 5-year overall survival in the TCGA (I-J), GSE31210 (K–L), GSE68465 (M–N) and GSE72094 (O–P). * *p* < 0.05; ** *p* < 0.01; *** *p* < 0.001; Figure S5 Ligand-receptor interactions between AHNAK2^+^ epithelial cells and other cells; Figure S6 Pseudotime analysis of epithelial cells. A: Trajectory color-coded by pseudotime (The color sequence from dark to light represents the evolution from early to late stages). B: Distribution of epithelial cells expressing AHNAK2 at high and low levels. C: AHNAK2 expression levels on different developmental trajectories (Blue to red indicate low to high expression levels.); Table S1: The baseline characteristics of the four datasets and primer sequences for quantitative RT-PCR.

## References

[1] Siegel R.L., Miller K.D., Fuchs H.E., Jemal A. (2022). Cancer statistics, 2022. CA A Cancer J. Clin..

[2] Sung H., Ferlay J., Siegel R.L., Laversanne M., Soerjomataram I., Jemal A., Bray F. (2021). Global Cancer Statistics 2020: Globocan Estimates of Incidence and Mortality Worldwide for 36 Cancers in 185 Countries. CA A Cancer J. Clin..

[3] Sun K., Li C., Liao S., Yao X., Ouyang Y., Liu Y., Wang Z., Li Z., Yao F. (2022). Ferritinophagy, a form of autophagic ferroptosis: New insights into cancer treatment. Front. Pharmacol..

[4] Jin X., Jiang C., Zou Z., Huang H., Li X., Xu S., Tan R. (2023). Ferritinophagy in the etiopathogenic mechanism of related diseases. J. Nutr. Biochem..

[5] Wang G., Li J., Zhu L., Zhou Z., Ma Z., Zhang H., Yang Y., Niu Q., Wang X. (2023). Identification of hepatocellular carcinoma-related subtypes and development of a prognostic model: A study based on ferritinophagy-related genes. Discover. Oncol..

[6] Dixon S.J., Lemberg K.M., Lamprecht M.R., Skouta R., Zaitsev E.M., Gleason C.E., Patel D.N., Bauer A.J., Cantley A.M., Yang W.S. (2012). Ferroptosis: An iron-dependent form of nonapoptotic cell death. Cell.

[7] Stockwell B.R., Friedmann Angeli J.P., Bayir H., Bush A.I., Conrad M., Dixon S.J., Fulda S., Gascón S., Hatzios S.K., Kagan V.E. (2017). Ferroptosis: A Regulated Cell Death Nexus Linking Metabolism, Redox Biology, and Disease. Cell.

[8] Hou W., Xie Y., Song X., Sun X., Lotze M.T., Zeh H.J., Kang R., Tang D. (2016). Autophagy promotes ferroptosis by degradation of ferritin. Autophagy.

[9] Tian Y., Lu J., Hao X., Li H., Zhang G., Liu X., Li X., Zhao C., Kuang W., Chen D. (2020). FTH1 Inhibits Ferroptosis Through Ferritinophagy in the 6-OHDA Model of Parkinson’s Disease. Neurother. J. Am. Soc. Exp. NeuroTherapeutics.

[10] Zardab M., Stasinos K., Grose R.P., Kocher H.M. (2022). The Obscure Potential of AHNAK2. Cancers.

[11] Wang D.W., Zheng H.Z., Cha N., Zhang X.J., Zheng M., Chen M.M., Tian L.X. (2020). Down-Regulation of AHNAK2 Inhibits Cell Proliferation, Migration and Invasion Through Inactivating the MAPK Pathway in Lung Adenocarcinoma. Technol. Cancer Res. Treat..

[12] Li X., Li H., Shao M.M., Miao J., Fu Y., Hu B. (2023). Downregulation of AHNAK2 inhibits cell cycle of lung adenocarcinoma cells by interacting with RUVBL1. Thorac. Cancer.

[13] Liu G., Guo Z., Zhang Q., Liu Z., Zhu D. (2020). AHNAK2 Promotes Migration, Invasion, and Epithelial-Mesenchymal Transition in Lung Adenocarcinoma Cells via the TGF-β/Smad3 Pathway. OncoTargets Ther..

[14] Cui Y., Liu X., Wu Y., Liang X., Dai J., Zhang Z., Guo R. (2022). Deleterious AHNAK2 Mutation as a Novel Biomarker for Immune Checkpoint Inhibitors in Non-Small Cell Lung Cancer. Front. Oncol..

[15] Okayama H., Kohno T., Ishii Y., Shimada Y., Shiraishi K., Iwakawa R., Furuta K., Tsuta K., Shibata T., Yamamoto S. (2012). Identification of genes upregulated in ALK-positive and EGFR/KRAS/ALK-negative lung adenocarcinomas. Cancer Res..

[16] Shedden K., Taylor J.M., Enkemann S.A., Tsao M.S., Yeatman T.J., Gerald W.L., Eschrich S., Jurisica I., Giordano T.J., Misek D.E. (2008). Gene expression-based survival prediction in lung adenocarcinoma: A multi-site, blinded validation study. Nat. Med..

[17] Schabath M.B., Welsh E.A., Fulp W.J., Chen L., Teer J.K., Thompson Z.J., Engel B.E., Xie M., Berglund A.E., Creelan B.C. (2016). Differential association of STK11 and TP53 with KRAS mutation-associated gene expression, proliferation and immune surveillance in lung adenocarcinoma. Oncogene.

[18] Davis S., Meltzer P.S. (2007). GEOquery: A bridge between the Gene Expression Omnibus (GEO) and BioConductor. Bioinformatics.

[19] Colaprico A., Silva T.C., Olsen C., Garofano L., Cava C., Garolini D., Sabedot T.S., Malta T.M., Pagnotta S.M., Castiglioni I. (2016). TCGAbiolinks: An R/Bioconductor package for integrative analysis of TCGA data. Nucleic Acids Res..

[20] Kim N., Kim H.K., Lee K., Hong Y., Cho J.H., Choi J.W., Lee J.I., Suh Y.L., Ku B.M., Eum H.H. (2020). Single-cell RNA sequencing demonstrates the molecular and cellular reprogramming of metastatic lung adenocarcinoma. Nat. Commun..

[21] Mayakonda A., Lin D.C., Assenov Y., Plass C., Koeffler H.P. (2018). Maftools: Efficient and comprehensive analysis of somatic variants in cancer. Genome Res..

[22] Zhang H., Meltzer P., Davis S. (2013). RCircos: An R package for Circos 2D track plots. BMC Bioinform..

[23] Harrow J., Frankish A., Gonzalez J.M., Tapanari E., Diekhans M., Kokocinski F., Aken B.L., Barrell D., Zadissa A., Searle S. (2012). GENCODE: The reference human genome annotation for The ENCODE Project. Genome Res..

[24] Hänzelmann S., Castelo R., Guinney J. (2013). GSVA: Gene set variation analysis for microarray and RNA-seq data. BMC Bioinform..

[25] Langfelder P., Horvath S. (2008). WGCNA: An R package for weighted correlation network analysis. BMC Bioinform..

[26] Wilkerson M.D., Hayes D.N. (2010). ConsensusClusterPlus: A class discovery tool with confidence assessments and item tracking. Bioinformatics.

[27] Love M.I., Huber W., Anders S. (2014). Moderated estimation of fold change and dispersion for RNA-seq data with DESeq2. Genome Biol..

[28] Blanche P., Dartigues J.F., Jacqmin-Gadda H. (2013). Estimating and comparing time-dependent areas under receiver operating characteristic curves for censored event times with competing risks. Stat. Med..

[29] Yu G., Wang L.G., Han Y., He Q.Y. (2012). clusterProfiler: An R package for comparing biological themes among gene clusters. Omics A J. Integr. Biol..

[30] Harris M.A., Clark J., Ireland A., Lomax J., Ashburner M., Foulger R., Eilbeck K., Lewis S., Marshall B., Mungall C. (2004). The Gene Ontology (GO) database and informatics resource. Nucleic Acids Res..

[31] Kanehisa M., Goto S. (2000). KEGG: Kyoto encyclopedia of genes and genomes. Nucleic Acids Res..

[32] Liberzon A., Subramanian A., Pinchback R., Thorvaldsdóttir H., Tamayo P., Mesirov J.P. (2011). Molecular signatures database (MSigDB) 3.0. Bioinformatics.

[33] Zeng D., Ye Z., Shen R., Yu G., Wu J., Xiong Y., Zhou R., Qiu W., Huang N., Sun L. (2021). IOBR: Multi-Omics Immuno-Oncology Biological Research to Decode Tumor Microenvironment and Signatures. Front. Immunol..

[34] Newman A.M., Liu C.L., Green M.R., Gentles A.J., Feng W., Xu Y., Hoang C.D., Diehn M., Alizadeh A.A. (2015). Robust enumeration of cell subsets from tissue expression profiles. Nat. Methods.

[35] Li T., Fan J., Wang B., Traugh N., Chen Q., Liu J.S., Li B., Liu X.S. (2017). TIMER: A Web Server for Comprehensive Analysis of Tumor-Infiltrating Immune Cells. Cancer Res..

[36] Aran D., Hu Z., Butte A.J. (2017). xCell: Digitally portraying the tissue cellular heterogeneity landscape. Genome Biol..

[37] Becht E., Giraldo N.A., Lacroix L., Buttard B., Elarouci N., Petitprez F., Selves J., Laurent-Puig P., Sautès-Fridman C., Fridman W.H. (2016). Estimating the population abundance of tissue-infiltrating immune and stromal cell populations using gene expression. Genome Biol..

[38] Yoshihara K., Shahmoradgoli M., Martínez E., Vegesna R., Kim H., Torres-Garcia W., Treviño V., Shen H., Laird P.W., Levine D.A. (2013). Inferring tumour purity and stromal and immune cell admixture from expression data. Nat. Commun..

[39] Racle J., de Jonge K., Baumgaertner P., Speiser D.E., Gfeller D. (2017). Simultaneous enumeration of cancer and immune cell types from bulk tumor gene expression data. eLife.

[40] Finotello F., Mayer C., Plattner C., Laschober G., Rieder D., Hackl H., Krogsdam A., Loncova Z., Posch W., Wilflingseder D. (2019). Molecular and pharmacological modulators of the tumor immune contexture revealed by deconvolution of RNA-seq data. Genome Med..

[41] Jiang P., Gu S., Pan D., Fu J., Sahu A., Hu X., Li Z., Traugh N., Bu X., Li B. (2018). Signatures of T cell dysfunction and exclusion predict cancer immunotherapy response. Nat. Med..

[42] Geeleher P., Cox N., Huang R.S. (2014). pRRophetic: An R package for prediction of clinical chemotherapeutic response from tumor gene expression levels. PLoS ONE.

[43] Trapnell C., Cacchiarelli D., Grimsby J., Pokharel P., Li S., Morse M., Lennon N.J., Livak K.J., Mikkelsen T.S., Rinn J.L. (2014). The dynamics and regulators of cell fate decisions are revealed by pseudotemporal ordering of single cells. Nat. Biotechnol..

[44] Jin S., Guerrero-Juarez C.F., Zhang L., Chang I., Ramos R., Kuan C.H., Myung P., Plikus M.V., Nie Q. (2021). Inference and analysis of cell-cell communication using CellChat. Nat. Commun..

[45] Chen X., Zhang T., Su W., Dou Z., Zhao D., Jin X., Lei H., Wang J., Xie X., Cheng B. (2022). Mutant p53 in cancer: From molecular mechanism to therapeutic modulation. Cell Death Dis..

[46] Lee S., Hwang N., Seok B.G., Lee S., Lee S.J., Chung S.W. (2023). Autophagy mediates an amplification loop during ferroptosis. Cell Death Dis..

[47] Zheng M., Liu J., Bian T., Liu L., Sun H., Zhou H., Zhao C., Yang Z., Shi J., Liu Y. (2021). Correlation between prognostic indicator AHNAK2 and immune infiltrates in lung adenocarcinoma. Int. Immunopharmacol..

[48] Zheng L., Li S., Zheng X., Guo R., Qu W. (2021). AHNAK2 is a novel prognostic marker and correlates with immune infiltration in papillary thyroid cancer: Evidence from integrated analysis. Int. Immunopharmacol..

[49] Koguchi D., Matsumoto K., Shimizu Y., Kobayashi M., Hirano S., Ikeda M., Sato Y., Iwamura M. (2021). Prognostic Impact of AHNAK2 Expression in Patients Treated with Radical Cystectomy. Cancers.

[50] Ye R., Liu D., Guan H., AiErken N., Fang Z., Shi Y., Zhang Y., Wang S. (2021). AHNAK2 promotes thyroid carcinoma progression by activating the NF-κB pathway. Life Sci..

[51] Xu M., Wen J., Xu Q., Li H., Lin B., Bhandari A., Qu J. (2022). AHNAK2 promotes the progression of differentiated thyroid cancer through PI3K/AKT signaling pathway. Curr. Cancer Drug Targets.

[52] Lin Q.Y., Qi Q.L., Hou S., Chen Z., Jiang N., Zhang L., Lin C.H. (2021). Silencing of AHNAK2 restricts thyroid carcinoma progression by inhibiting the Wnt/β-catenin pathway. Neoplasma.

[53] Wang Z., Liu T., Xue W., Fang Y., Chen X., Xu L., Zhang L., Guan K., Pan J., Zheng L. (2020). ARNTL2 promotes pancreatic ductal adenocarcinoma progression through TGF/BETA pathway and is regulated by miR-26a-5p. Cell Death Dis..

[54] Zhang H., Shan G., Jin X., Yu X., Bi G., Feng M., Wang H., Lin M., Zhan C., Wang Q. (2022). ARNTL2 is an indicator of poor prognosis, promotes epithelial-to-mesenchymal transition and inhibits ferroptosis in lung adenocarcinoma. Transl. Oncol..

[55] Wang T., Wang K., Zhu X., Chen N. (2023). ARNTL2 upregulation of ACOT7 promotes NSCLC cell proliferation through inhibition of apoptosis and ferroptosis. BMC Mol. Cell Biol..

[56] Buchan S.L., Rogel A., Al-Shamkhani A. (2018). The immunobiology of CD27 and OX40 and their potential as targets for cancer immunotherapy. Blood.

[57] Starzer A.M., Berghoff A.S. (2020). New emerging targets in cancer immunotherapy: CD27 (TNFRSF7). ESMO Open.

[58] Borelli A., Irla M. (2021). Lymphotoxin: From the physiology to the regeneration of the thymic function. Cell Death Differ..

[59] Legut M., Gajic Z., Guarino M., Daniloski Z., Rahman J.A., Xue X., Lu C., Lu L., Mimitou E.P., Hao S. (2022). A genome-scale screen for synthetic drivers of T cell proliferation. Nature.

[60] Wu P., Guo Y. (2022). Susceptibility Loci in SLC15A1, UGT1A3, and CWC27 Genes Associated with Bladder Cancer in the Northeast Chinese Population. BioMed Res. Int..

[61] Zhang Y., Fan Q., Guo Y., Zhu K. (2020). Eight-gene signature predicts recurrence in lung adenocarcinoma. Cancer Biomark. Sect. A Dis. Markers.

[62] Shangguan C., Gan G., Zhang J., Wu J., Miao Y., Zhang M., Li B., Mi J. (2018). Cancer-associated fibroblasts enhance tumor (18)F-FDG uptake and contribute to the intratumor heterogeneity of PET-CT. Theranostics.

[63] Shen C., Xuan B., Yan T., Ma Y., Xu P., Tian X., Zhang X., Cao Y., Ma D., Zhu X. (2020). m(6)A-dependent glycolysis enhances colorectal cancer progression. Mol. Cancer.

[64] Liu X.S., Yang J.W., Zeng J., Chen X.Q., Gao Y., Kui X.Y., Liu X.Y., Zhang Y., Zhang Y.H., Pei Z.J. (2022). SLC2A1 is a Diagnostic Biomarker Involved in Immune Infiltration of Colorectal Cancer and Associated With m6A Modification and ceRNA. Front. Cell Dev. Biol..

[65] Liu G., Zeng X., Wu B., Zhao J., Pan Y. (2020). RNA-Seq analysis of peripheral blood mononuclear cells reveals unique transcriptional signatures associated with radiotherapy response of nasopharyngeal carcinoma and prognosis of head and neck cancer. Cancer Biol. Ther..

[66] Evans C.S., Ruhl D.A., Chapman E.R. (2015). An Engineered Metal Sensor Tunes the Kinetics of Synaptic Transmission. J. Neurosci. Off. J. Soc. Neurosci..

[67] Li L., Liu H., Krout M., Richmond J.E., Wang Y., Bai J., Weeratunga S., Collins B.M., Ventimiglia D., Yu Y. (2021). A novel dual Ca^2+^ sensor system regulates Ca^2+^-dependent neurotransmitter release. J. Cell Biol..

