# Supplementary material for: Identification of a Novel Signature Based on Ferritinophagy-Related Genes to Predict Prognosis in Lung Adenocarcinoma: Focus on AHNAK2
Source: Bioengineering (Basel). 2024 Oct 26;11(11):1070. doi: 10.3390/bioengineering11111070 (PMC11591153; doi:10.3390/bioengineering11111070)
Supplement: Supplementary file 1 [file bioengineering-11-01070-s001.zip › FigureS1-S6.pdf]

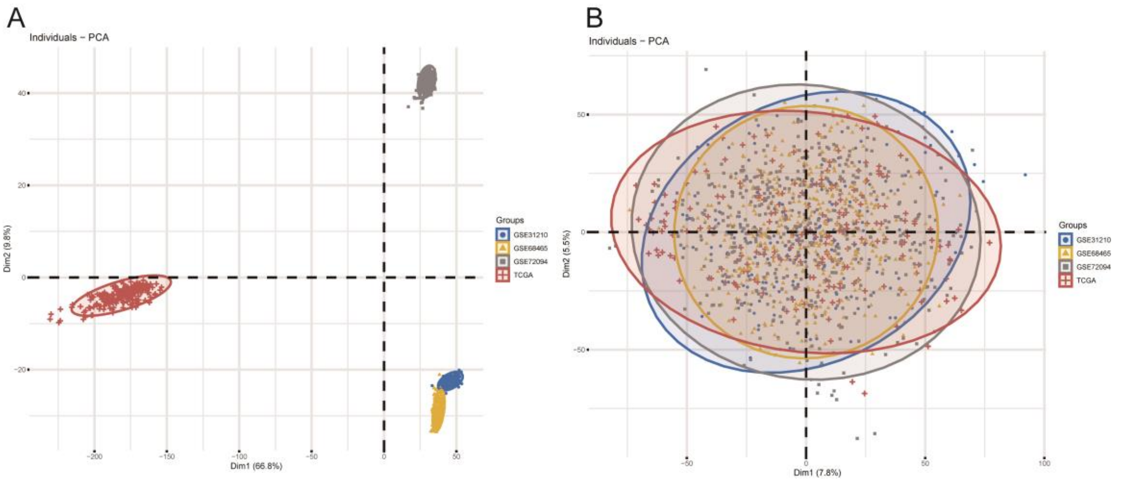

Figure S1. Batch effect plot of the dataset. A: PCA representation of the dataset distribution before data correction. B: PCA representation of the dataset distribution after data correction.

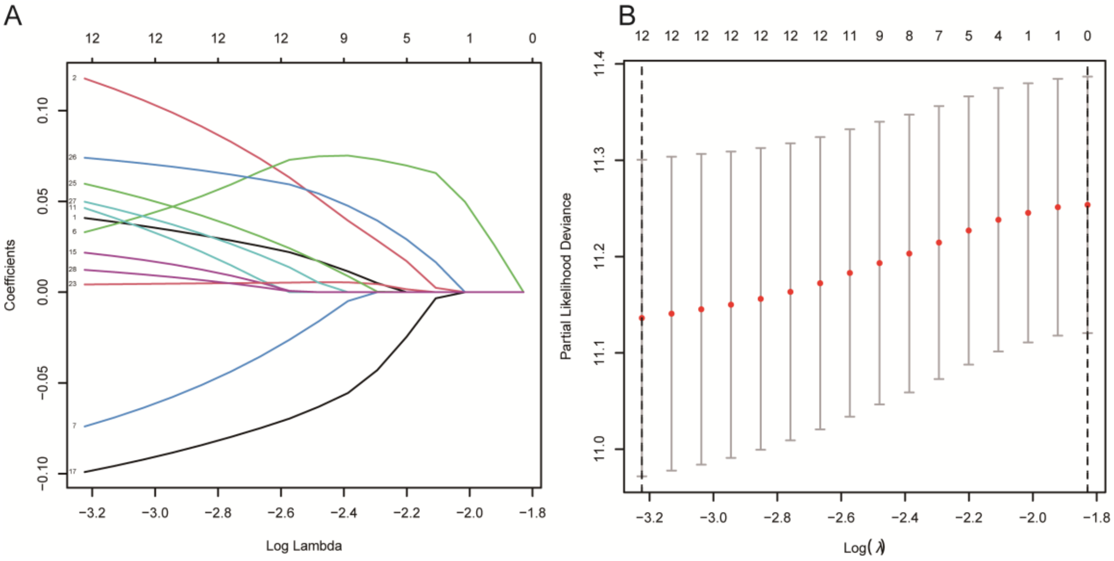

Figure S2 LASSO regression model for constructing prognostic gene signatures. A: Distribution of LASSO coefficients. B: The generated coefficient distribution plots.

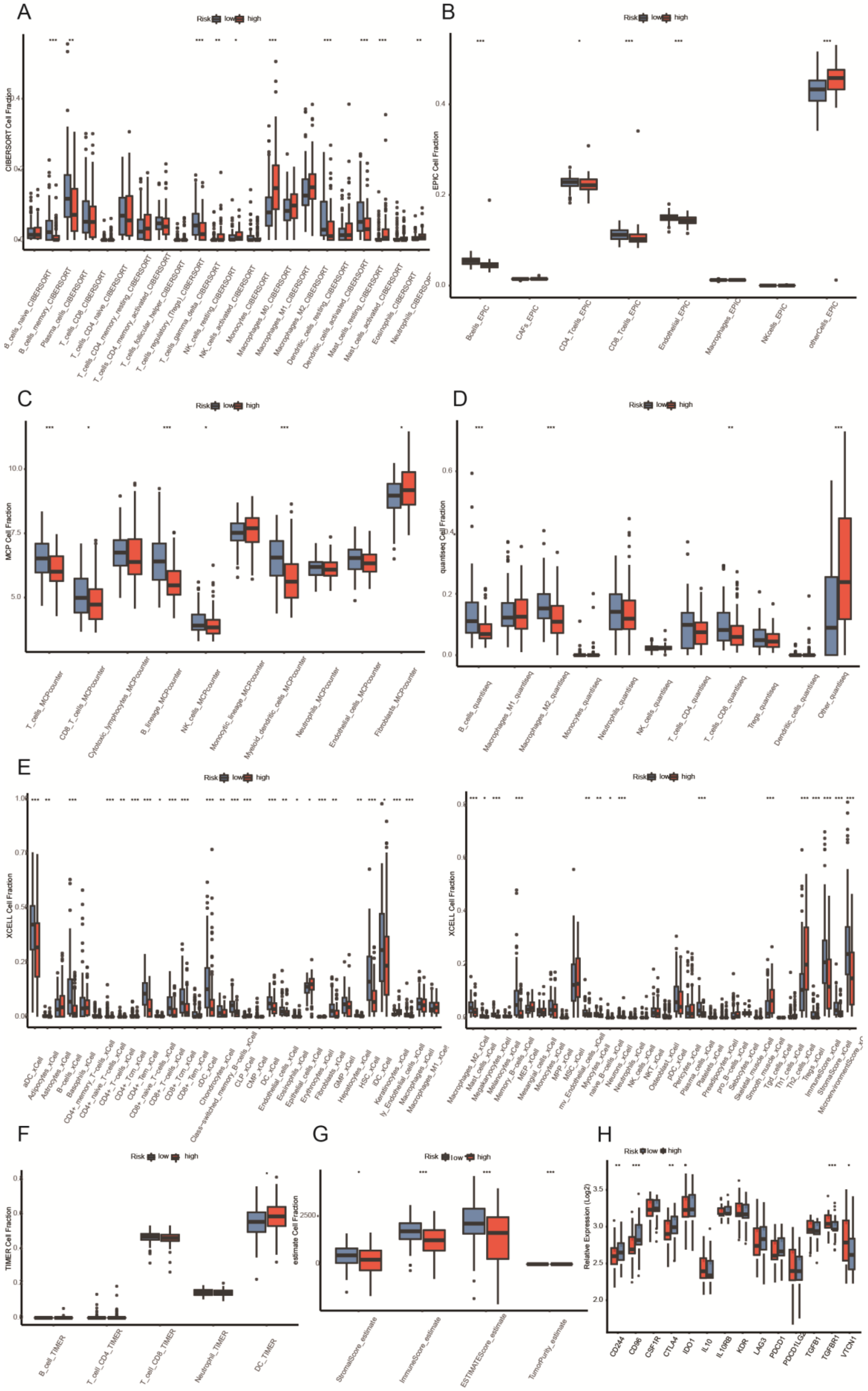
A
Risk low high
CIBERSORT Cell Fraction
B_cells_naive_CIBERSORT
B_cells_memory_CIBERSORT
Plasma_cells_CIBERSORT
T_cells_CD8_CIBERSORT
T_cells_CD4_naive_CIBERSORT
T_cells_CD4_memory_resting_CIBERSORT
T_cells_CD4_memory_activated_CIBERSORT
T_cells_follicular_helper_CIBERSORT
T_cells_regulatory_(Tregs)_CIBERSORT
T_cells_gamma_delta_CIBERSORT
NK_cells_resting_CIBERSORT
NK_cells_activated_CIBERSORT
Monocytes_CIBERSORT
Macrophages_M0_CIBERSORT
Macrophages_M1_CIBERSORT
Macrophages_M2_CIBERSORT
Dendritic_cells_resting_CIBERSORT
Dendritic_cells_activated_CIBERSORT
Mast_cells_resting_CIBERSORT
Mast_cells_activated_CIBERSORT
Eosinophils_CIBERSORT
B
Risk low high
EPIC Cell Fraction
Bcells_EPIC
CAFs_EPIC
CD4_Tcells_EPIC
CD8_Tcells_EPIC
Endothelial_EPIC
Macrophages_EPIC
NKcells_EPIC
otherCells_EPIC
C
Risk low high
MCP Cell Fraction
T_cells_MCPcounter
CD8_T_cells_MCPcounter
Cytotoxic_lymphocytes_MCPcounter
B_lineage_MCPcounter
NK_cells_MCPcounter
Monocytic_lineage_MCPcounter
Myeloid_dendritic_cells_MCPcounter
Neutrophils_MCPcounter
Endothelial_cells_MCPcounter
Fibroblasts_MCPcounter
D
Risk low high
quantiseq Cell Fraction
B_cells_quantiseq
Macrophages_M1_quantiseq
Macrophages_M2_quantiseq
Monocytes_quantiseq
Neutrophils_quantiseq
NK_cells_quantiseq
T_cells_CD4_quantiseq
T_cells_CD8_quantiseq
Tregs_quantiseq
Dendritic_cells_quantiseq
Other_quantiseq
E
Risk low high
XCELL Cell Fraction
F
Risk low high
TIMER Cell Fraction
B_cell_TIMER
T_cell_CD4_TIMER
T_cell_CD8_TIMER
Neutrophil_TIMER
DC_TIMER
G
Risk low high
estimate Cell Fraction
StromalScore_estimate
ImmuneScore_estimate
ESTIMATEScore_estimate
TumorPurity_estimate
H
Risk low high
Relative Expression (Log2)
CD244
CD96
CSF1R
CTLA4
IDO1
IL10
IL10RB
KDR
LAG3
PDCD1
PDCD1LG2
TGFB1
TGFBR1
VTCN1

Figure S3 Immune analysis. Box plot showing the difference between the low and high risk groups using the CIBERSORT (A), EPIC (B), MCP_counter (C), Quanti-seq (D), xCell (E), and TIMER (F) algorithm. G: Stromal score, immune score, ESTIMATE score, and tumor purity of the low and high risk groups. H: Box plot showing the difference between the low and high risk groups in terms of immune checkpoint. *p < 0.05; **p < 0.01; ***p < 0.001.

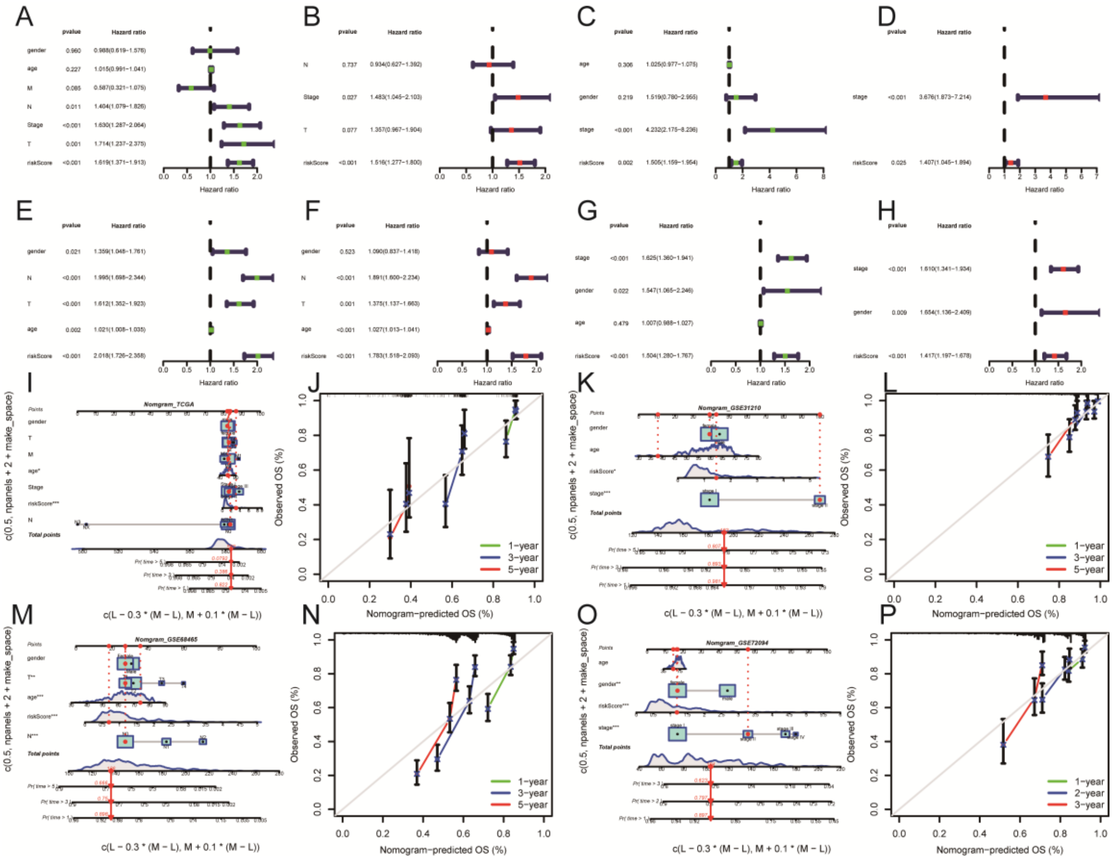

Figure S4 Independent prognostic analysis of risk score. Forest plot showing the univariate and multivariate Cox regression analysis of risk score and clinical phenotype in the TCGA (A-B), GSE31210 (C-D), GSE68465 (E-F) and GSE72094 (G-H). Kaplan-Meier plot for predicting 1-year, 3-year, and 5-year overall survival in the TCGA (I-J), GSE31210 (K-L), GSE68465 (M-N) and GSE72094 (O-P). *P<0.05; **P<0.01;***P<0.001.

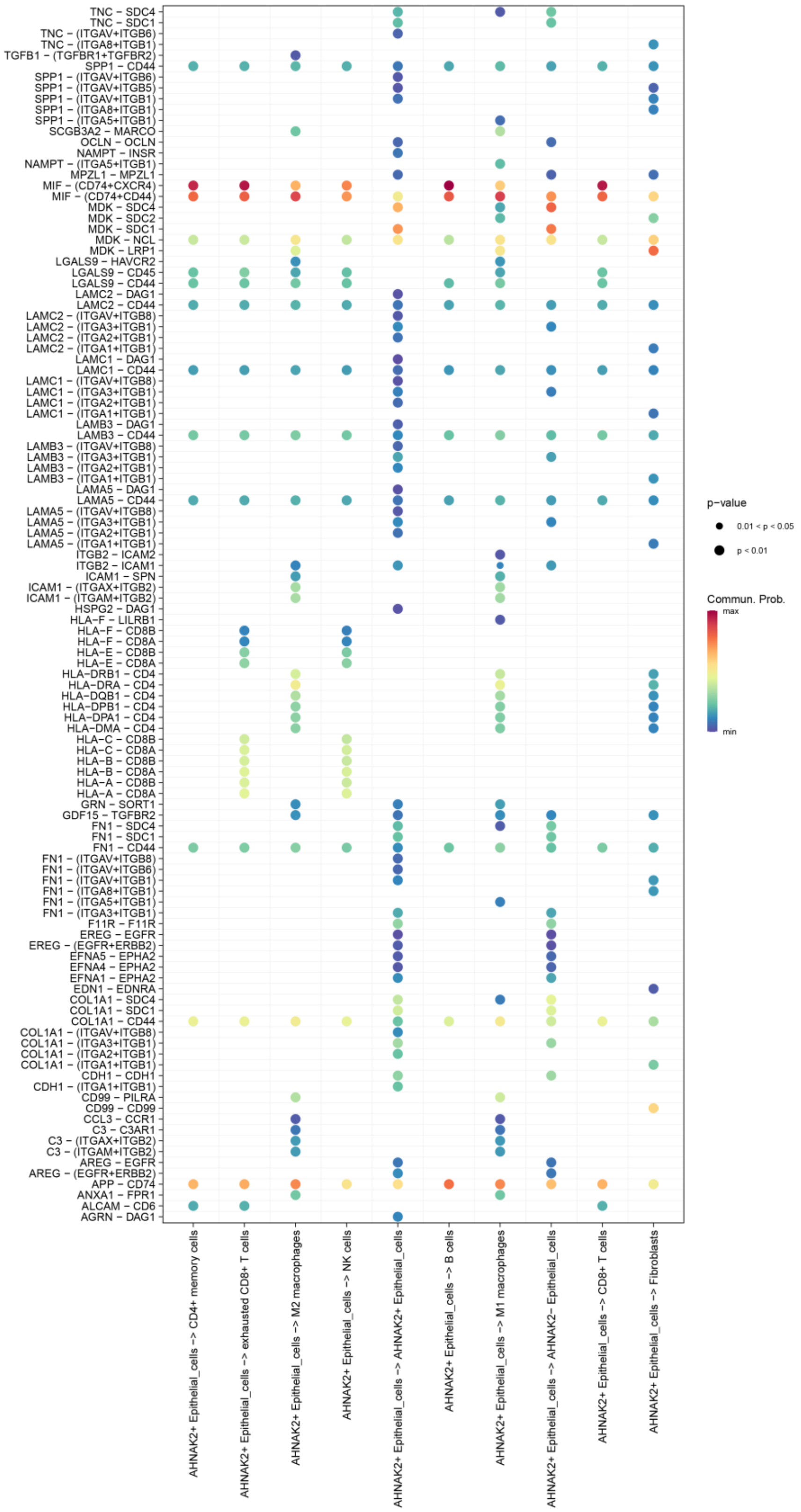

Figure S5 Ligand-receptor interactions between AHNAK2^{+} epithelial cells and other cells.

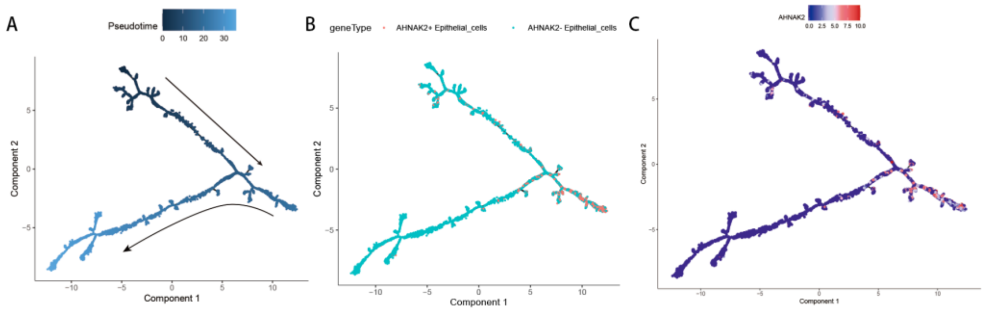

Figure S6 Pseudotime analysis of epithelial cells. A: Trajectory color-coded by pseudotime (The color sequence from dark to light represents the evolution from early to late stages). B: Distribution of epithelial cells expressing AHNAK2 at high and low levels. C: AHNAK2 expression levels on different developmental trajectories (Blue to red indicate low to high expression levels.).
